# Supplementary material for: Direct Interaction Between CD34+ Hematopoietic Stem Cells and Mesenchymal Stem Cells Reciprocally Preserves Stemness
Source: Cancers (Basel). 2024 Nov 27;16(23):3972. doi: 10.3390/cancers16233972 (PMC11640414; doi:10.3390/cancers16233972)
Supplement: Supplementary file 1 [file cancers-16-03972-s001.zip › Supplementary Table 1.pdf]

Supplementary Table 1: Source of $CD34^+$ cells.

Type of leukemia	*%CD34$^+$*	*%CD34$^+$/CD38$^-$/CD45$^-$*
T-cell Lymphoma	1.1	0.8
	1.3	0.7
Hodgkin Lymphoma	0.5	0.2
	0.4	0.3
	2.7	2.5
Non-Hodgkin Lymphoma	0.2	0.1
	0.4	0.3
Lymphoma	1.6	1.4
Multiple Myeloma	4	2.2
	2	1.2
Ewing Sarcoma	0.4	0.2
Neuroblastoma	0.2	0.1
