# Supplementary material for: Direct Interaction Between CD34+ Hematopoietic Stem Cells and Mesenchymal Stem Cells Reciprocally Preserves Stemness
Source: Cancers (Basel). 2024 Nov 27;16(23):3972. doi: 10.3390/cancers16233972 (PMC11640414; doi:10.3390/cancers16233972)
Supplement: Supplementary file 1 [file cancers-16-03972-s001.zip › Supplementary Table 2.pdf]

Supplementary Table 2: Primary AML cells used in the study.

Patient number	*Age*	*Sex*	*Diagnotic/relpase*	*FAB subtype*	*Source*	*Molecular/ Cytogenetics*
AML 1	76	Female	Relapse	M2	Peripheral blood	FLT3 mutation/46,XX
AML 2	95	Female	Diagnostic	M2	Peripheral blood	ND
AML 3	67	Female	Diagnostic	M5	Peripheral blood	NPM1 mutation/46,XX

ND: not determined.
