# Supplementary figures and images for: Direct Interaction Between CD34+ Hematopoietic Stem Cells and Mesenchymal Stem Cells Reciprocally Preserves Stemness
Source: Cancers (Basel). 2024 Nov 27;16(23):3972. doi: 10.3390/cancers16233972 (PMC11640414; doi:10.3390/cancers16233972)

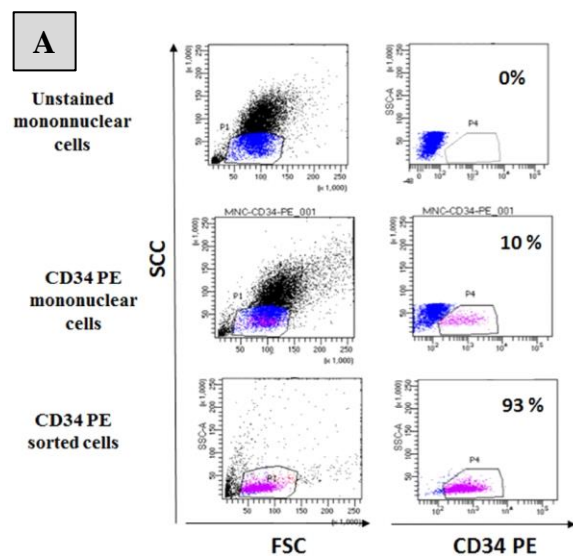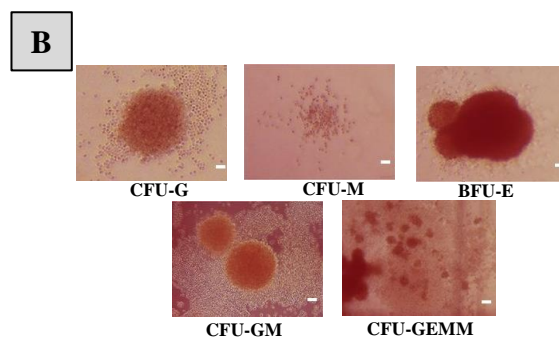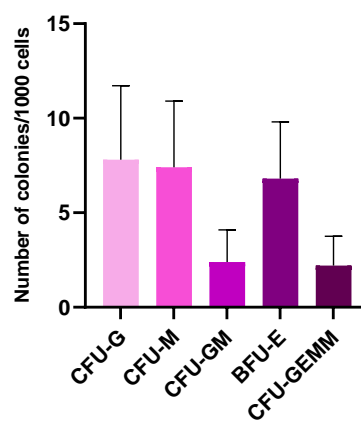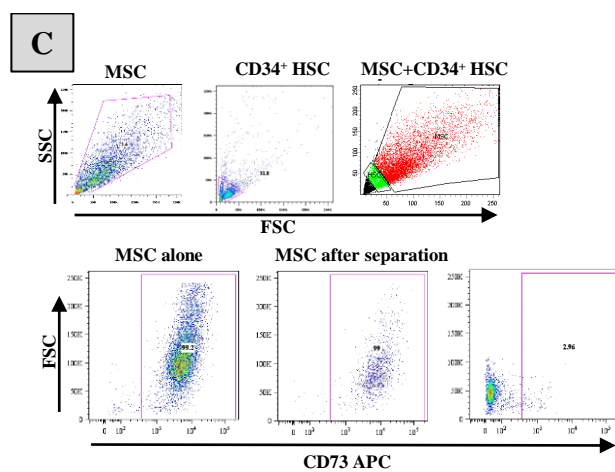

Supplement: Supplementary file 1 [file cancers-16-03972-s001.zip › Supplementary Figure 1.pdf]

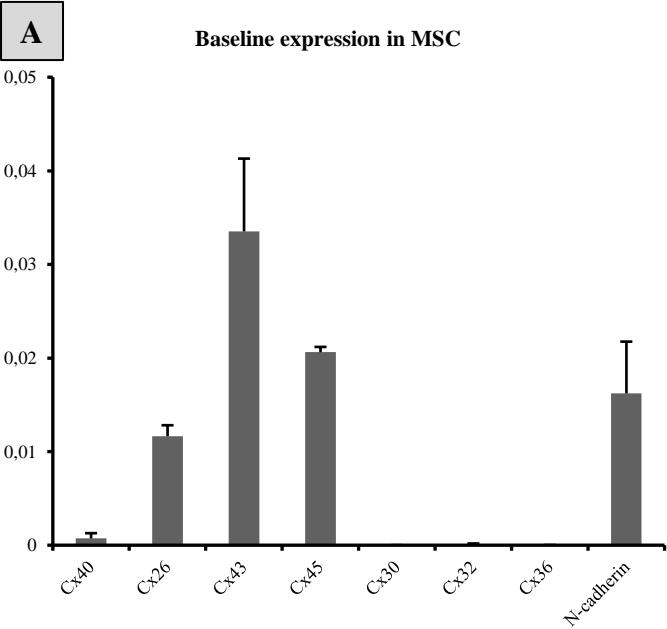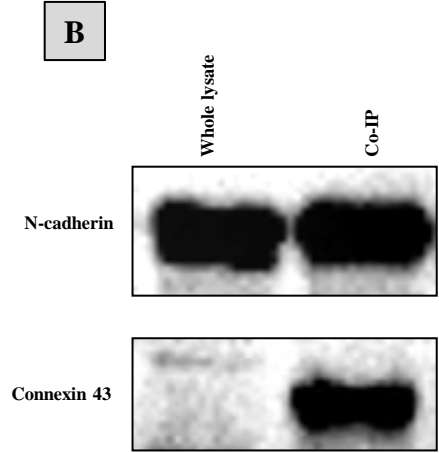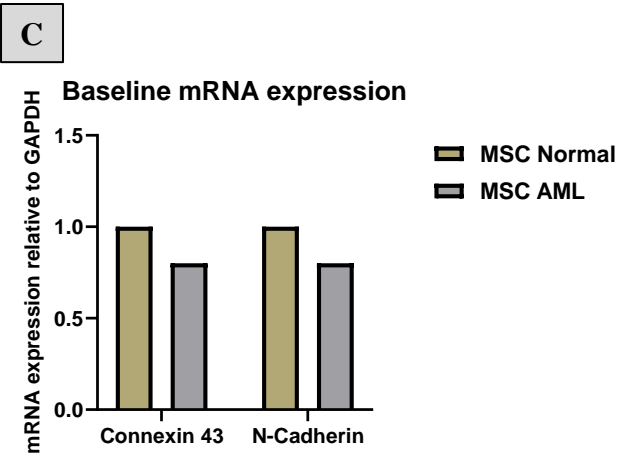

Supplement: Supplementary file 1 [file cancers-16-03972-s001.zip › Supplementary Figure 2.pdf]
